# Crystal structure of bis[bis(4-azaniumylphenyl) sulfone] tetranitrate monohydrate

**DOI:** 10.1107/S2056989017014803

**Published:** 2017-10-20

**Authors:** Amani Hind Benahsene, Lamia Bendjeddou, Hocine Merazig

**Affiliations:** a Unité de Recherche Chimie de l’Environnement et Moléculaire, Structurale ‘CHEMS’, Faculté des Sciences Exactes,Campus Chaabet Ersas, Université Frères Mentouri Constantine 1, 25000 Constantine, Algeria

**Keywords:** dapsone, 4,4′-di­phenyl­sulfones, crystal structure, protonation, hydrogen-bonding, nitrate salt

## Abstract

In the title compound, the hydrated tetra­(nitrate) salt of dapsone (4,4′-di­amino­diphenyl­sulfone), 2C_12_H_14_N_2_O_2_S^2+^·4NO_3_^−^·H_2_O {alternative name: bis[bis­(4,4′-di­aza­niumylphen­yl) sulfone] tetra­nitrate monohydrate}, the cations are conformationally similar, with comparable dihedral angles between the two benzene rings in each of 70.03 (18) and 69.69 (19)°. In the crystal, mixed cation–anion–water mol­ecule layers lying parallel to the (001) plane are formed through N—H⋯O, O—H⋯O and C—H⋯O hydrogen-bonding inter­actions and these layers are further extended into an overall three-dimensional supra­molecular network structure. Inter-ring π–π inter­actions are also present [minimum ring centroid separation = 3.693 (3) Å].

## Abstract 2

The synthesis and structural determination of bis­(4,4′-di­ammonio­diphen­yl)sulfone tetra­(nitrate) monohydrate is reported. The crystal structure features N—H⋯O, O—H⋯O and C—H⋯O hydrogen-bonds and π–π inter­actions.

## Chemical context   

Dapsone (4,4′-di­amino­diphenyl­sulfone), a very weak Lewis base (p*K*a *ca* 2), is a drug that has been used to treat a diversity of diseases including tuberculosis, leprosy, malaria and AIDS-related pneumonia (Wilson *et al.*, 1991 ▸). The crystal structure of dapsone was first reported in 1970 (Dickenson *et al.*, 1970 ▸) and redetermined a number of times (Bocelli & Cantoni, 1990 ▸; Su *et al.*, 1992 ▸; Bertolasi *et al.*, 1993 ▸). The structure of its partial (0.33) hydrate has also been determined (Kus’mina *et al.*, 1981 ▸; Bel’skii *et al.*, 1983 ▸). To the best of our knowledge there are no reported polymorphic forms of dapsone.

Sulfones are good hydrogen-bond acceptors since their ability to participate as such in hydrogen-bonding inter­actions is increased by the highly polar nature of the sulfur–oxygen bond (Almarsson & Zaworotko, 2004 ▸; Eccles *et al.*, 2010 ▸). In order to enrich the knowledge of such kinds of compound and to investigate the effect of hydrogen bonding on the chemical and structural features, we report here the synthesis and crystal structure analysis of a new salt of dapsone, the hydrated dinitrate 2C_12_H_14_N_2_O_2_S^2+^·4NO_3_^−^·H_2_O. In terms of other compounds containing the ammonio-substituted dapsone cation species, only the mono-ammonio–dapsone salt 4-(4-amino­phenyl­sulfon­yl)anilinium 2-carb­oxy-4,6-di­nitro­phen­olate monohydrate has been reported (Smith & Wermuth, 2013 ▸). Surprisingly, the literature has not revealed any other crystal structure containing the (4,4′-di­ammonio)-substituted di­phenyl­sulfone.
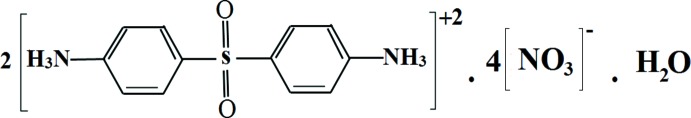


## Structural commentary   

**Figure 1:**
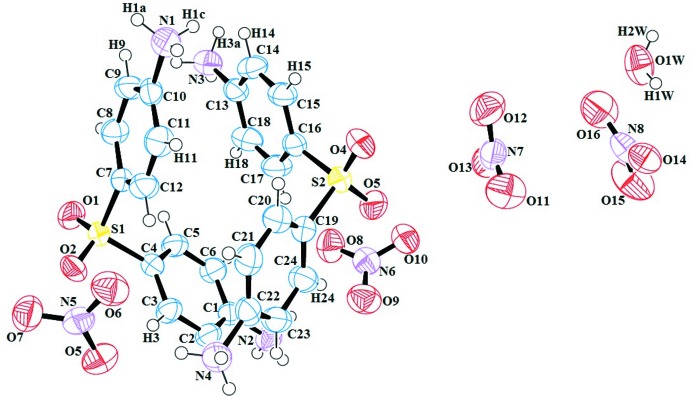
The asymmetric unit of the title compound, showing the atom-numbering scheme for the two cations (*A*, left and *B*, right), the four nitrate anions and the water mol­ecule of solvation. Displacement ellipsoids are drawn at the 50% probability level and H atoms are shown as spheres of arbitrary radii.

The title compound crystallizes in the ortho­rhom­bic space group *P*2_1_2_1_2_1_ with two (4,4′-di­ammonio­diphen­yl)sulfone cations (*A* and *B*), four nitrate anions and one water mol­ecule (O1*W*) in the asymmetric unit (Fig. 1 ▸). The di­amino­diphenyl­sulfone unit is protonated at both N1 and N2 in *A* and N3 and N4 in *B*. The two cations are conformationally similar with the dihedral angles between the benzene rings of the anilinic moieties of cation *A* [defined by (N2/C1–C6) (*Aa*) and (N1/C7–C12) (*Ab*)] and cation *B* [defined by (N3/C13–C18) (*Ba*) and (N4/ C19–C24) (*Bb*)] are 70.03 (18) and 69.69 (19)°, respectively. As expected the anilinium groups are planar with maximum r.m.s. deviations of 0.0044, 0.0120, 0.0114 and 0.0072 Å, respectively.

## Supra­molecular features   

**Figure 2:**
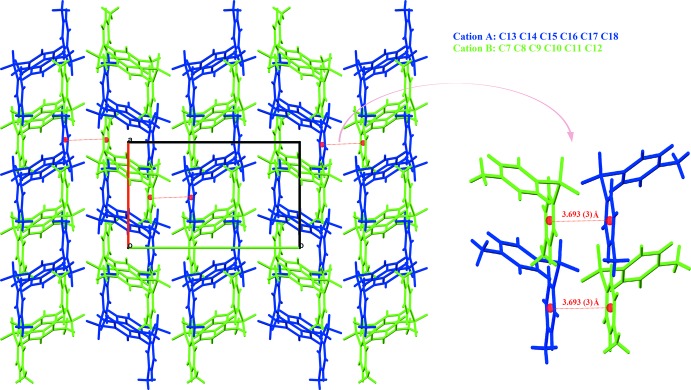
Part of the crystal structure, showing double cationic chains and π–π associations, with nitrate anions and the water mol­ecule omitted.

**Figure 3:**
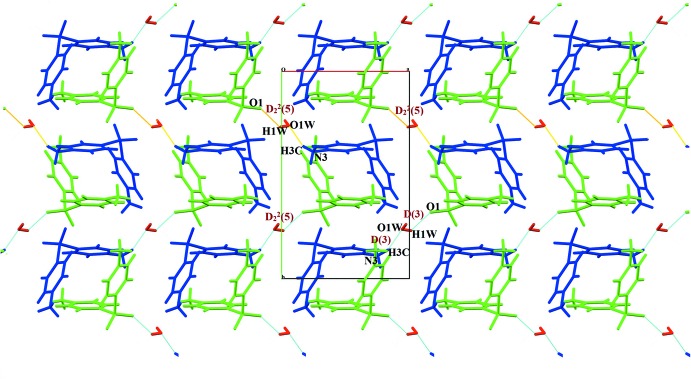
Part of the crystal structure, with nitrate anions omitted, showing the dual role of the water mol­ecule in hydrogen bonding (dashed lines) and aggregation of *D*(3) and *D* ^2^_2_(5) motifs *via* O—H⋯O and N—H⋯O inter­actions.

**Figure 4:**
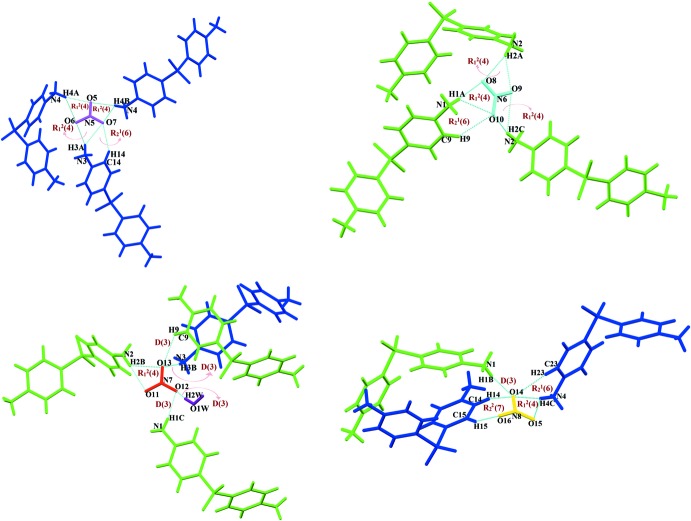
Hydrogen-bond inter­actions around each nitrate anion and aggregation of 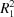
(4), 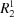
(6), 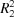
(7) and *D*(3) motifs.

**Figure 5:**
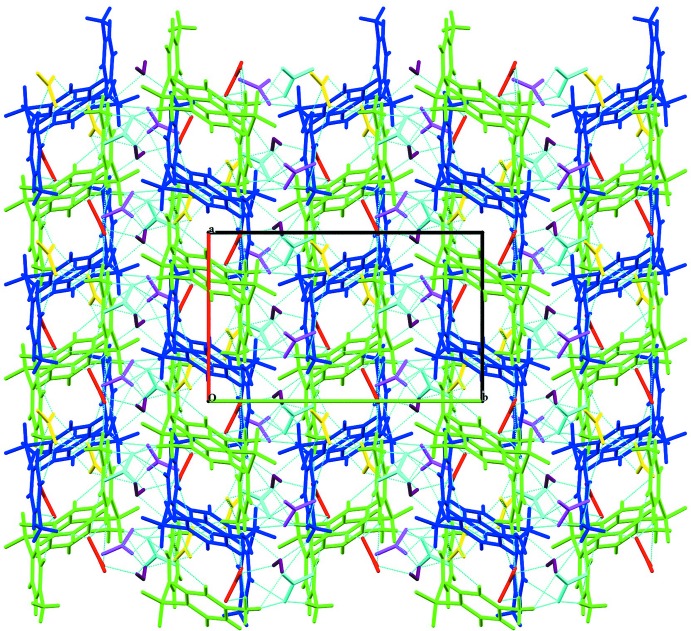
The overall crystal packing in the three-dimensional structure in the unit cell viewed along *c*. The red lines represent nitrate anions.

The hydrogen-bonded supra­molecular assembly in the crystal of the title compound is generated by a total of 28 independent inter­actions, dominated by anilinium N—H⋯O hydrogen bonds involving only nitro-O acceptors and a single water acceptor, but no sulfone O atoms are involved (Table 1 ▸). The water mol­ecule forms two hydrogen bonds, to sulfone O1^vi^ and nitro O12^vii^ acceptors. The two cations *A* and *B* are associated through π–π inter­actions [ring centroid separation *CgAb*⋯*CgBa* ^i^ = 3.693 (3) Å [symmetry code: (i) −*x* + 1, *y* + 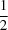, −*z* + 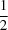] and form double cationic chain sub-structures that extend along the *a*-axis direction (Fig. 2 ▸). The water mol­ecule O1*W*, which plays a dual role as both donor and acceptor in hydrogen-bonding inter­actions, bridges the cations *via* one sulfonyl group (Fig. 3 ▸) and also bridges one nitro group, giving the combination of the hydrogen-bond sequence N3—H⋯O1*W*/O1*W*—H⋯O1, involving a *D* ^2^_2_(5) bond motif (Fig. 3 ▸). The cations and anions are inter­linked by the ammonio N—H⋯O(nitro) hydrogen bonds through rings and finite chains involving 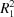
(4), 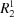
(6), 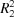
(7) and *D*(3) motifs (Fig. 4 ▸), generating a three-dimensional hydrogen-bonded network structure in which a number of C—H⋯O(nitro) inter­actions are also found (Fig. 5 ▸).

## Database survey   

A search of the Cambridge Structural Database (Version 5.38; Groom *et al.*, 2016 ▸) shows 20 hits concerning the 4,4′-di­amino­diphenyl sulfone. Only one containing a protonated dapsone species, the mono-cationic (4-ammonio-4′-amino-diphen­yl)sulfone, a phenolate (Smith & Wermuth, 2013 ▸).

## Synthesis and crystallization   

Fe(NO_3_)_3_·9H_2_O (20.19 mg, 0.50 mmol) in EtOH (2 ml) was added dropwise to 4,4′-di­amino­diphenyl sulfone (12.41 mg, 0.50 mmol) in EtOH (5 ml), with continuous stirring at room temperature for 72 h. Slow evaporation of this solution yielded yellow crystals suitable for X-ray analysis within 5 d.

## Refinement   

Crystal data, data collection and structure refinement details are summarized in Table 2 ▸. The aromatic H atoms were placed at calculated positions with C—H fixed at 0.93 Å and *U*_iso_(H) = 1.2*U*_eq_(C). All N—H atoms were located by difference methods but were subsequently restrained in the refinement with N—H = 0.89 Å and *U*_iso_ = 1.2*U*_eq_(N). The H atoms of the water mol­ecule were also located in a Fourier map and were allowed to ride with a restrained O—H bond length = 0.85 (1) Å and H⋯H = 1.39 (2) Å and *U*_iso_(H) = 1.5 *U*_eq_(O). Although not of relevance in this achiral compound, the Flack absolute structure parameter (Flack, 1983 ▸) was determined as 0.02 (9) for 4494 Friedel pairs.

## Supplementary Material

Crystal structure: contains datablock(s) I. DOI: 10.1107/S2056989017014803/zs2390sup1.cif

Structure factors: contains datablock(s) I. DOI: 10.1107/S2056989017014803/zs2390Isup2.hkl

CCDC reference: 1579678

Additional supporting information:  crystallographic information; 3D view; checkCIF report

## Figures and Tables

**Table 1:** Hydrogen-bond geometry (Å, °)

*D*—H⋯*A*	*D*—H	H⋯*A*	*D*⋯*A*	*D*—H⋯*A*
N1—H1*A*⋯O8^i^	0.89	2.0800	2.959 (4)	169
N1—H1*A*⋯O10^i^	0.89	2.4400	3.057 (4)	127
N1—H1*B*⋯O14^ii^	0.89	1.9300	2.796 (4)	163
N1—H1*C*⋯O12^ii^	0.89	2.0500	2.920 (5)	166
N2—H2*A*⋯O8	0.89	2.2800	3.132 (4)	160
N2—H2*A*⋯O9	0.89	2.3300	2.937 (5)	125
N2—H2*B*⋯O11^iii^	0.89	2.1300	3.015 (5)	176
N2—H2*B*⋯O13^iii^	0.89	2.3900	2.979 (4)	124
N2—H2*C*⋯O9^iii^	0.89	2.5600	3.086 (5)	119
N2—H2*C*⋯O10^iii^	0.89	2.0400	2.912 (5)	166
N3—H3*A*⋯O6^iv^	0.89	2.1000	2.957 (4)	161
N3—H3*A*⋯O7^iv^	0.89	2.3700	3.115 (5)	141
N3—H3*B*⋯O13^i^	0.89	2.0500	2.906 (4)	163
N3—H3*C*⋯O1*W* ^i^	0.89	1.9100	2.740 (5)	154
N4—H4*A*⋯O5	0.89	2.1000	2.982 (4)	169
N4—H4*A*⋯O6	0.89	2.3900	3.071 (4)	134
N4—H4*B*⋯O5^v^	0.89	2.0800	2.960 (5)	171
N4—H4*B*⋯O7^v^	0.89	2.3800	2.988 (5)	125
N4—H4*C*⋯O14^iii^	0.89	1.9800	2.861 (4)	169
N4—H4*C*⋯O15^iii^	0.89	2.5000	3.111 (5)	126
O1*W*—H1*W*⋯O1^vi^	0.86 (4)	2.18 (5)	2.834 (5)	133 (5)
O1*W*—H2*W*⋯O12^vii^	0.85 (4)	2.19 (6)	2.940 (6)	147 (5)
C9—H9⋯O10^i^	0.93	2.6000	3.359 (5)	139
C9—H9⋯O13^i^	0.93	2.5700	3.206 (5)	126
C14—H14⋯O7^iv^	0.93	2.5000	3.295 (5)	143
C14—H14⋯O14^ii^	0.93	2.5300	3.194 (5)	128
C15—H15⋯O16^ii^	0.93	2.4600	3.351 (6)	160
C23—H23⋯O14^iii^	0.93	2.5200	3.227 (4)	133

Symmetry codes: (i) 
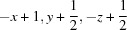
; (ii) 
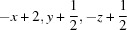
; (iii) 
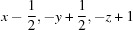
; (iv) 
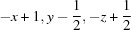
; (v) 
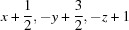
; (vi) 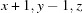; (vii) 
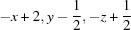
.

**Table 2:** Experimental details

Crystal data	
Chemical formula	2C_12_H_14_N_2_O_2_S^2+^·4NO_3_^−^·H_2_O
*M*_r_	766.68
Crystal system, space group	Orthorhombic, *P*2_1_2_1_2_1_
Temperature (K)	293
*a*, *b*, *c* (Å)	9.366 (5), 15.203 (5), 23.070 (5)
*V* (Å^3^)	3285.1 (2)
*Z*	4
Radiation type	Mo *K*α
μ (mm^−1^)	0.25
Crystal size (mm)	0.1 × 0.04 × 0.03

Data collection	
Diffractometer	Bruker APEXII CCD
Absorption correction	–
No. of measured, independent and observed [*I* > 2σ(*I*)] reflections	22152, 9858, 5434
*R*_int_	0.045
(sin θ/λ)_max_ (Å^−1^)	0.715

Refinement	
*R*[*F* ^2^ > 2σ(*F* ^2^)], *wR*(*F* ^2^), *S*	0.063, 0.201, 0.98
No. of reflections	9858
No. of parameters	466
No. of restraints	3
Δρ_max_, Δρ_min_ (e Å^−3^)	0.94, −0.29
Absolute structure	(Flack, 1983 ▸), 4494 Friedel pairs
Absolute structure parameter	0.02 (9)

Computer programs: *APEX2* (Bruker, 2006 ▸), *SAINT* (Bruker, 2006 ▸), *SHELXS97* (Sheldrick, 2008 ▸), *SHELXL97* (Sheldrick, 2008 ▸) within *WinGX* (Farrugia, 2012 ▸), *ORTEP-3 for Windows* (Farrugia, 2012 ▸), *Mercury* (Macrae *et al.*, 2006 ▸) and *POVRay* (Persistence of Vision, 2004 ▸).

## supplementary crystallographic information

### Crystal data

**Table:**

2C_12_H_14_N_2_O_2_S^2+^·4NO_3_^−^·H_2_O	*F*(000) = 1592
*M**_r_* = 766.68	*D*_x_ = 1.55 Mg m^−^^3^
Orthorhombic, *P*2_1_2_1_2_1_	Mo *K*α radiation, λ = 0.71073 Å
Hall symbol: P 2ac 2ab	Cell parameters from 4292 reflections
*a* = 9.366 (5) Å	θ = 2.8–30.6°
*b* = 15.203 (5) Å	µ = 0.25 mm^−^^1^
*c* = 23.070 (5) Å	*T* = 293 K
*V* = 3285.1 (2) Å^3^	Prism, yellow
*Z* = 4	0.1 × 0.04 × 0.03 mm

### Data collection

**Table:**

Bruker APEXII CCD diffractometer	5434 reflections with *I* > 2σ(*I*)
Radiation source: fine-focus sealed tube	*R*_int_ = 0.045
Graphite monochromator	θ_max_ = 30.6°, θ_min_ = 2.8°
φ and ω scans	*h* = −13→10
22152 measured reflections	*k* = −20→21
9858 independent reflections	*l* = −20→32

### Refinement

**Table:**

Refinement on *F*^2^	Secondary atom site location: difference Fourier map
Least-squares matrix: full	Hydrogen site location: inferred from neighbouring sites
*R*[*F*^2^ > 2σ(*F*^2^)] = 0.063	*w* = 1/[σ^2^(*F*_o_^2^) + (0.1089*P*)^2^] where *P* = (*F*_o_^2^ + 2*F*_c_^2^)/3
*wR*(*F*^2^) = 0.201	(Δ/σ)_max_ < 0.001
*S* = 0.98	Δρ_max_ = 0.94 e Å^−^^3^
9858 reflections	Δρ_min_ = −0.29 e Å^−^^3^
466 parameters	Absolute structure: (Flack, 1983), 4494 Friedel pairs
3 restraints	Absolute structure parameter: 0.02 (9)
Primary atom site location: structure-invariant direct methods	

### Special details

**Table:**

Geometry. All esds (except the esd in the dihedral angle between two l.s. planes) are estimated using the full covariance matrix. The cell esds are taken into account individually in the estimation of esds in distances, angles and torsion angles; correlations between esds in cell parameters are only used when they are defined by crystal symmetry. An approximate (isotropic) treatment of cell esds is used for estimating esds involving l.s. planes.
Refinement. Refinement of F^2^ against ALL reflections. The weighted R-factor wR and goodness of fit S are based on F^2^, conventional R-factors R are based on F, with F set to zero for negative F^2^. The threshold expression of F^2^ > 2sigma(F^2^) is used only for calculating R-factors(gt) etc. and is not relevant to the choice of reflections for refinement. R-factors based on F^2^ are statistically about twice as large as those based on F, and R- factors based on ALL data will be even larger.

### Fractional atomic coordinates and isotropic or equivalent isotropic displacement parameters (Å^2^)

**Table:**

	*x*	*y*	*z*	*U*_iso_*/*U*_eq_	
S1	0.30537 (10)	0.66966 (6)	0.36491 (3)	0.0460 (2)	
S2	0.73567 (13)	0.33251 (6)	0.34908 (4)	0.0573 (3)	
O1	0.1674 (3)	0.6792 (2)	0.33905 (11)	0.0662 (8)	
O2	0.3663 (4)	0.74142 (16)	0.39709 (11)	0.0641 (8)	
N1	0.7194 (4)	0.6046 (2)	0.17307 (13)	0.0632 (9)	
N2	0.2628 (4)	0.3644 (2)	0.52443 (12)	0.0534 (8)	
C1	0.2741 (4)	0.4383 (2)	0.48528 (13)	0.0438 (8)	
C2	0.2055 (5)	0.4351 (2)	0.43285 (15)	0.0526 (11)	
C3	0.2172 (4)	0.5057 (2)	0.39507 (14)	0.0509 (13)	
O3	0.6919 (4)	0.25896 (17)	0.38437 (12)	0.0774 (10)	
C4	0.2981 (4)	0.5777 (2)	0.41134 (13)	0.0424 (7)	
O4	0.8628 (4)	0.3279 (2)	0.31386 (12)	0.0797 (10)	
C5	0.3660 (4)	0.5804 (2)	0.46487 (14)	0.0483 (8)	
C6	0.3540 (4)	0.5102 (2)	0.50192 (14)	0.0485 (8)	
C7	0.4267 (4)	0.6429 (2)	0.30900 (13)	0.0390 (7)	
C8	0.3757 (4)	0.6264 (2)	0.25382 (14)	0.0486 (8)	
C9	0.4746 (4)	0.6132 (3)	0.20947 (15)	0.0530 (9)	
C10	0.6172 (4)	0.6166 (2)	0.22081 (15)	0.0449 (8)	
C11	0.6665 (4)	0.6310 (3)	0.27560 (16)	0.0571 (10)	
C12	0.5709 (5)	0.6446 (3)	0.32016 (16)	0.0558 (12)	
N3	0.2464 (4)	0.3877 (2)	0.18676 (13)	0.0581 (8)	
N4	0.8110 (4)	0.6370 (2)	0.50927 (13)	0.0579 (8)	
O5	0.5968 (3)	0.7800 (2)	0.49317 (12)	0.0697 (8)	
O6	0.7370 (4)	0.7782 (2)	0.41897 (13)	0.0752 (9)	
O7	0.5916 (4)	0.88673 (19)	0.43116 (13)	0.0716 (8)	
C13	0.3656 (5)	0.3788 (2)	0.22676 (15)	0.0501 (9)	
C14	0.5035 (5)	0.3837 (3)	0.20635 (15)	0.0554 (10)	
C15	0.6167 (5)	0.3731 (2)	0.24417 (15)	0.0528 (9)	
C16	0.5899 (5)	0.3571 (2)	0.30186 (14)	0.0484 (9)	
C17	0.4495 (5)	0.3514 (3)	0.32251 (15)	0.0636 (11)	
C18	0.3379 (5)	0.3635 (3)	0.28489 (17)	0.0648 (11)	
C19	0.7558 (4)	0.4240 (2)	0.39558 (14)	0.0503 (9)	
C20	0.8352 (5)	0.4962 (3)	0.37815 (16)	0.0585 (10)	
C21	0.8536 (5)	0.5659 (3)	0.41473 (16)	0.0580 (10)	
C22	0.7930 (4)	0.5624 (2)	0.46966 (16)	0.0491 (8)	
C23	0.7158 (5)	0.4897 (3)	0.48763 (15)	0.0553 (10)	
C24	0.6959 (5)	0.4201 (3)	0.45057 (15)	0.0560 (9)	
N5	0.6416 (4)	0.8159 (2)	0.44782 (13)	0.0550 (8)	
O8	0.3116 (4)	0.2214 (2)	0.42874 (15)	0.0777 (12)	
O9	0.4742 (4)	0.2291 (2)	0.49463 (14)	0.0799 (9)	
O10	0.4700 (3)	0.1205 (2)	0.43431 (13)	0.0703 (8)	
N6	0.4179 (4)	0.1904 (2)	0.45266 (14)	0.0563 (8)	
O11	1.0082 (4)	0.1199 (3)	0.39162 (15)	0.0863 (14)	
O12	0.9768 (4)	0.1153 (3)	0.29935 (14)	0.0909 (11)	
O13	0.8139 (3)	0.0628 (2)	0.35749 (13)	0.0666 (7)	
N7	0.9327 (4)	0.0995 (3)	0.34960 (16)	0.0635 (9)	
O14	1.2484 (3)	−0.05892 (19)	0.38079 (11)	0.0620 (7)	
O15	1.0556 (4)	−0.1246 (3)	0.40490 (17)	0.1024 (13)	
O16	1.0780 (4)	−0.0673 (3)	0.32060 (17)	0.0936 (11)	
N8	1.1241 (4)	−0.0855 (2)	0.36797 (18)	0.0632 (9)	
O1W	0.9435 (4)	−0.2379 (3)	0.27703 (17)	0.0861 (10)	
H1A	0.69946	0.64248	0.14476	0.0760*	
H1B	0.71292	0.54993	0.15959	0.0760*	
H1C	0.80755	0.61427	0.18596	0.0760*	
H2A	0.26874	0.31454	0.50439	0.0640*	
H2B	0.33346	0.36669	0.55019	0.0640*	
H2C	0.17936	0.36658	0.54281	0.0640*	
H3	0.15161	0.38603	0.42270	0.0630*	
H4	0.17140	0.50466	0.35932	0.0612*	
H6	0.41919	0.62942	0.47551	0.0580*	
H7	0.39929	0.51103	0.53780	0.0580*	
H8	0.27809	0.62423	0.24647	0.0580*	
H9	0.44306	0.60197	0.17197	0.0640*	
H11	0.76413	0.63164	0.28288	0.0690*	
H12	0.60380	0.65478	0.35759	0.0670*	
H3A	0.26860	0.36256	0.15311	0.0700*	
H3B	0.22780	0.44444	0.18109	0.0700*	
H3C	0.16968	0.36146	0.20164	0.0700*	
H4A	0.75103	0.67985	0.49938	0.0690*	
H4B	0.90036	0.65667	0.50711	0.0690*	
H4C	0.79262	0.61977	0.54538	0.0690*	
H14	0.52045	0.39426	0.16724	0.0660*	
H15	0.71014	0.37657	0.23074	0.0630*	
H17	0.43220	0.33958	0.36142	0.0760*	
H18	0.24430	0.36140	0.29828	0.0780*	
H20	0.87605	0.49724	0.34142	0.0700*	
H21	0.90570	0.61489	0.40308	0.0690*	
H23	0.67743	0.48804	0.52479	0.0660*	
H24	0.64326	0.37130	0.46210	0.0670*	
H1W	1.012 (4)	−0.232 (4)	0.3016 (18)	0.1290*	
H2W	0.974 (6)	−0.262 (4)	0.2464 (14)	0.1290*	

### Atomic displacement parameters (Å^2^)

**Table:**

	*U*^11^	*U*^22^	*U*^33^	*U*^12^	*U*^13^	*U*^23^
S1	0.0579 (6)	0.0387 (4)	0.0416 (4)	0.0132 (4)	0.0042 (4)	0.0004 (3)
S2	0.0848 (8)	0.0408 (4)	0.0462 (4)	0.0166 (5)	0.0009 (5)	−0.0036 (4)
O1	0.063 (2)	0.0803 (19)	0.0551 (14)	0.0293 (16)	0.0019 (13)	0.0090 (14)
O2	0.106 (2)	0.0354 (12)	0.0515 (14)	0.0031 (14)	0.0146 (15)	−0.0086 (11)
N1	0.066 (2)	0.066 (2)	0.0571 (17)	0.0138 (18)	0.0114 (17)	0.0058 (16)
N2	0.057 (2)	0.0562 (17)	0.0471 (15)	−0.0021 (15)	0.0056 (14)	0.0091 (14)
C1	0.048 (2)	0.0424 (17)	0.0409 (16)	0.0046 (15)	0.0074 (14)	0.0033 (13)
C2	0.060 (2)	0.0470 (19)	0.0507 (18)	−0.0112 (18)	−0.0043 (18)	−0.0008 (16)
C3	0.061 (3)	0.053 (2)	0.0388 (16)	−0.0028 (18)	−0.0073 (16)	−0.0036 (15)
O3	0.138 (3)	0.0358 (13)	0.0580 (15)	0.0072 (16)	−0.0079 (19)	0.0059 (12)
C4	0.046 (2)	0.0422 (17)	0.0393 (15)	0.0050 (15)	0.0010 (14)	−0.0039 (13)
O4	0.095 (2)	0.081 (2)	0.0631 (15)	0.0410 (19)	0.0049 (16)	−0.0148 (16)
C5	0.053 (2)	0.0477 (19)	0.0444 (17)	−0.0042 (16)	−0.0104 (16)	0.0013 (15)
C6	0.055 (2)	0.052 (2)	0.0379 (16)	−0.0008 (17)	−0.0070 (15)	0.0032 (15)
C7	0.0455 (19)	0.0329 (15)	0.0386 (15)	0.0020 (13)	−0.0010 (13)	0.0015 (12)
C8	0.041 (2)	0.060 (2)	0.0454 (17)	0.0019 (17)	−0.0036 (15)	0.0012 (16)
C9	0.058 (3)	0.064 (2)	0.0377 (16)	0.0023 (19)	−0.0045 (16)	−0.0064 (16)
C10	0.044 (2)	0.0390 (17)	0.0517 (19)	0.0063 (15)	0.0092 (16)	0.0058 (14)
C11	0.044 (2)	0.066 (2)	0.061 (2)	0.0043 (19)	−0.0061 (17)	−0.0006 (19)
C12	0.057 (2)	0.065 (2)	0.0452 (17)	0.0061 (19)	−0.0100 (17)	−0.0074 (17)
N3	0.061 (2)	0.063 (2)	0.0504 (15)	−0.0046 (17)	0.0062 (16)	−0.0037 (14)
N4	0.060 (2)	0.0552 (17)	0.0588 (17)	0.0040 (16)	−0.0192 (16)	−0.0112 (15)
O5	0.071 (2)	0.084 (2)	0.0544 (15)	−0.0047 (17)	0.0026 (15)	0.0124 (15)
O6	0.065 (2)	0.088 (2)	0.0731 (19)	0.0049 (18)	0.0114 (16)	−0.0076 (16)
O7	0.084 (2)	0.0550 (17)	0.0759 (18)	0.0000 (16)	−0.0108 (18)	0.0090 (15)
C13	0.066 (2)	0.0405 (18)	0.0444 (17)	−0.0001 (17)	0.0082 (17)	−0.0025 (14)
C14	0.070 (3)	0.059 (2)	0.0377 (16)	−0.011 (2)	0.0049 (18)	0.0057 (16)
C15	0.062 (2)	0.055 (2)	0.0418 (17)	−0.0037 (19)	0.0119 (17)	−0.0011 (16)
C16	0.073 (3)	0.0311 (15)	0.0410 (16)	0.0004 (16)	0.0041 (17)	−0.0022 (13)
C17	0.083 (3)	0.069 (3)	0.0386 (17)	0.000 (2)	0.0142 (19)	0.0015 (18)
C18	0.071 (3)	0.074 (3)	0.050 (2)	−0.001 (2)	0.014 (2)	−0.0085 (19)
C19	0.059 (2)	0.0466 (18)	0.0456 (17)	0.0111 (18)	−0.0043 (17)	−0.0001 (15)
C20	0.070 (3)	0.055 (2)	0.0510 (19)	0.005 (2)	0.0057 (19)	−0.0003 (17)
C21	0.063 (3)	0.048 (2)	0.063 (2)	0.0006 (19)	−0.0015 (19)	0.0048 (18)
C22	0.044 (2)	0.0480 (19)	0.0555 (19)	0.0057 (16)	−0.0094 (17)	−0.0065 (16)
C23	0.067 (3)	0.056 (2)	0.0435 (18)	−0.0021 (19)	0.0001 (17)	−0.0053 (16)
C24	0.065 (3)	0.053 (2)	0.0502 (19)	−0.0029 (19)	0.0025 (18)	−0.0056 (16)
N5	0.058 (2)	0.063 (2)	0.0449 (15)	−0.0029 (17)	−0.0085 (15)	−0.0002 (15)
O8	0.066 (2)	0.073 (2)	0.094 (2)	0.0072 (17)	−0.0080 (19)	0.0010 (17)
O9	0.076 (2)	0.090 (2)	0.0737 (18)	−0.0019 (19)	−0.0081 (18)	−0.0174 (18)
O10	0.069 (2)	0.0683 (19)	0.0733 (17)	0.0112 (16)	0.0057 (16)	−0.0074 (15)
N6	0.052 (2)	0.057 (2)	0.0606 (18)	−0.0048 (16)	0.0093 (16)	0.0007 (16)
O11	0.063 (2)	0.110 (3)	0.086 (2)	−0.012 (2)	−0.0061 (19)	−0.013 (2)
O12	0.082 (2)	0.117 (3)	0.0738 (19)	0.017 (2)	0.0103 (18)	0.0278 (19)
O13	0.0531 (18)	0.0745 (19)	0.0723 (17)	−0.0014 (15)	−0.0003 (15)	0.0061 (15)
N7	0.064 (2)	0.064 (2)	0.063 (2)	0.0171 (19)	0.0051 (19)	0.0120 (18)
O14	0.0541 (18)	0.0684 (17)	0.0635 (16)	−0.0024 (15)	0.0120 (14)	−0.0060 (14)
O15	0.096 (3)	0.082 (2)	0.130 (3)	−0.024 (2)	0.054 (3)	−0.012 (2)
O16	0.066 (2)	0.116 (3)	0.099 (2)	0.007 (2)	−0.014 (2)	−0.026 (2)
N8	0.054 (2)	0.058 (2)	0.078 (2)	0.0040 (17)	0.014 (2)	−0.0212 (19)
O1W	0.057 (2)	0.077 (2)	0.124 (3)	0.0099 (17)	−0.007 (2)	−0.011 (2)

### Geometric parameters (Å, º)

**Table:**

S1—O1	1.431 (3)	C8—C7	1.383 (5)
S1—O2	1.438 (3)	C8—C9	1.395 (5)
S1—C4	1.762 (3)	C8—H8	0.93
S1—C7	1.766 (3)	N7—O12	1.254 (5)
S2—O3	1.443 (3)	C22—C23	1.384 (6)
S2—O4	1.444 (4)	C22—C21	1.390 (5)
S2—C19	1.766 (4)	C20—C21	1.366 (5)
S2—C16	1.787 (4)	C20—C19	1.386 (5)
O13—N7	1.258 (5)	C20—H20	0.93
O14—N8	1.267 (5)	O16—N8	1.207 (5)
O5—N5	1.253 (4)	C4—C3	1.383 (5)
N1—C10	1.470 (5)	C13—C14	1.376 (6)
N1—H1B	0.89	C13—C18	1.386 (5)
N1—H1C	0.89	C13—N3	1.455 (5)
N1—H1A	0.89	C3—H4	0.93
O9—N6	1.250 (4)	C14—C15	1.383 (6)
N2—C1	1.445 (4)	C14—H14	0.93
N2—H2A	0.89	C15—C16	1.376 (5)
N2—H2C	0.89	C15—H15	0.93
N2—H2B	0.89	C16—C17	1.401 (6)
O6—N5	1.253 (4)	C24—C23	1.373 (5)
O7—N5	1.235 (4)	C24—C19	1.388 (5)
O10—N6	1.243 (4)	C24—H24	0.93
N4—C22	1.466 (5)	N8—O15	1.221 (5)
N4—H4A	0.89	C9—C10	1.362 (6)
N4—H4B	0.89	C9—H9	0.93
N4—H4C	0.89	C12—C7	1.375 (5)
O8—N6	1.233 (5)	C12—C11	1.379 (6)
O1W—H2W	0.848 (10)	C12—H12	0.93
O1W—H1W	0.859 (10)	C23—H23	0.93
C1—C2	1.371 (5)	C10—C11	1.363 (5)
C1—C6	1.379 (5)	C21—H21	0.93
O11—N7	1.239 (5)	C11—H11	0.93
C2—C3	1.388 (5)	C17—C18	1.371 (6)
C2—H3	0.93	C17—H17	0.93
C5—C6	1.372 (5)	C18—H18	0.93
C5—C4	1.389 (5)	N3—H3C	0.89
C5—H6	0.93	N3—H3A	0.89
C6—H7	0.93	N3—H3B	0.89
			
O1—S1—O2	119.81 (19)	C14—C13—C18	121.1 (4)
O1—S1—C4	107.38 (19)	C14—C13—N3	119.9 (3)
O2—S1—C4	107.65 (15)	C18—C13—N3	119.0 (4)
O1—S1—C7	107.45 (16)	C4—C3—C2	119.1 (3)
O2—S1—C7	107.26 (17)	C4—C3—H4	120.5
C4—S1—C7	106.61 (15)	C2—C3—H4	120.5
O3—S2—O4	120.9 (2)	C13—C14—C15	119.8 (3)
O3—S2—C19	107.32 (16)	C13—C14—H14	120.1
O4—S2—C19	107.0 (2)	C15—C14—H14	120.1
O3—S2—C16	106.8 (2)	C16—C15—C14	119.4 (4)
O4—S2—C16	107.32 (17)	C16—C15—H15	120.3
C19—S2—C16	106.67 (17)	C14—C15—H15	120.3
C10—N1—H1B	109.5	C15—C16—C17	120.7 (4)
C10—N1—H1C	109.5	C15—C16—S2	119.1 (3)
H1B—N1—H1C	109.5	C17—C16—S2	119.8 (3)
C10—N1—H1A	109.5	C23—C24—C19	118.8 (4)
H1B—N1—H1A	109.5	C23—C24—H24	120.6
H1C—N1—H1A	109.5	C19—C24—H24	120.6
C1—N2—H2A	109.5	O16—N8—O15	123.7 (5)
C1—N2—H2C	109.5	O16—N8—O14	117.9 (4)
H2A—N2—H2C	109.5	O15—N8—O14	118.4 (4)
C1—N2—H2B	109.5	C10—C9—C8	120.3 (3)
H2A—N2—H2B	109.5	C10—C9—H9	119.8
H2C—N2—H2B	109.5	C8—C9—H9	119.8
C22—N4—H4A	109.5	C7—C12—C11	119.7 (3)
C22—N4—H4B	109.5	C7—C12—H12	120.2
H4A—N4—H4B	109.5	C11—C12—H12	120.2
C22—N4—H4C	109.5	C12—C7—C8	121.0 (3)
H4A—N4—H4C	109.5	C12—C7—S1	119.4 (3)
H4B—N4—H4C	109.5	C8—C7—S1	119.5 (3)
H2W—O1W—H1W	111 (3)	C24—C23—C22	120.0 (3)
C2—C1—C6	121.9 (3)	C24—C23—H23	120
C2—C1—N2	119.3 (3)	C22—C23—H23	120
C6—C1—N2	118.8 (3)	O8—N6—O10	119.5 (4)
C1—C2—C3	119.3 (3)	O8—N6—O9	120.5 (4)
C1—C2—H3	120.4	O10—N6—O9	120.0 (4)
C3—C2—H3	120.4	C20—C19—C24	121.0 (3)
O7—N5—O5	120.9 (4)	C20—C19—S2	120.3 (3)
O7—N5—O6	120.3 (4)	C24—C19—S2	118.6 (3)
O5—N5—O6	118.8 (4)	C9—C10—C11	121.1 (3)
C6—C5—C4	119.6 (3)	C9—C10—N1	119.3 (3)
C6—C5—H6	120.2	C11—C10—N1	119.6 (3)
C4—C5—H6	120.2	C20—C21—C22	118.8 (4)
C5—C6—C1	119.1 (3)	C20—C21—H21	120.6
C5—C6—H7	120.4	C22—C21—H21	120.6
C1—C6—H7	120.4	C10—C11—C12	119.7 (4)
C7—C8—C9	118.1 (4)	C10—C11—H11	120.1
C7—C8—H8	120.9	C12—C11—H11	120.1
C9—C8—H8	120.9	C18—C17—C16	119.5 (3)
O11—N7—O12	119.2 (4)	C18—C17—H17	120.3
O11—N7—O13	120.2 (4)	C16—C17—H17	120.3
O12—N7—O13	120.6 (4)	C17—C18—C13	119.5 (4)
C23—C22—C21	121.1 (3)	C17—C18—H18	120.3
C23—C22—N4	119.4 (3)	C13—C18—H18	120.3
C21—C22—N4	119.5 (4)	C13—N3—H3C	109.5
C21—C20—C19	120.2 (4)	C13—N3—H3A	109.5
C21—C20—H20	119.9	H3C—N3—H3A	109.5
C19—C20—H20	119.9	C13—N3—H3B	109.5
C3—C4—C5	121.0 (3)	H3C—N3—H3B	109.5
C3—C4—S1	118.9 (3)	H3A—N3—H3B	109.5
C5—C4—S1	120.0 (3)		
			
C6—C1—C2—C3	0.4 (6)	O1—S1—C7—C12	169.1 (3)
N2—C1—C2—C3	−179.5 (3)	O2—S1—C7—C12	39.0 (3)
C4—C5—C6—C1	−0.3 (6)	C4—S1—C7—C12	−76.0 (3)
C2—C1—C6—C5	−0.3 (6)	O1—S1—C7—C8	−6.5 (3)
N2—C1—C6—C5	179.6 (3)	O2—S1—C7—C8	−136.6 (3)
C6—C5—C4—C3	0.7 (6)	C4—S1—C7—C8	108.3 (3)
C6—C5—C4—S1	176.9 (3)	C19—C24—C23—C22	0.8 (6)
O1—S1—C4—C3	36.4 (3)	C21—C22—C23—C24	−1.2 (6)
O2—S1—C4—C3	166.7 (3)	N4—C22—C23—C24	178.8 (4)
C7—S1—C4—C3	−78.5 (3)	C21—C20—C19—C24	−1.2 (6)
O1—S1—C4—C5	−139.8 (3)	C21—C20—C19—S2	−178.4 (3)
O2—S1—C4—C5	−9.6 (4)	C23—C24—C19—C20	0.4 (6)
C7—S1—C4—C5	105.3 (3)	C23—C24—C19—S2	177.6 (3)
C5—C4—C3—C2	−0.6 (6)	O3—S2—C19—C20	161.2 (3)
S1—C4—C3—C2	−176.8 (3)	O4—S2—C19—C20	30.0 (4)
C1—C2—C3—C4	0.0 (6)	C16—S2—C19—C20	−84.6 (4)
C18—C13—C14—C15	0.2 (6)	O3—S2—C19—C24	−16.0 (4)
N3—C13—C14—C15	−178.4 (3)	O4—S2—C19—C24	−147.2 (3)
C13—C14—C15—C16	0.3 (6)	C16—S2—C19—C24	98.2 (3)
C14—C15—C16—C17	0.2 (6)	C8—C9—C10—C11	1.5 (6)
C14—C15—C16—S2	173.8 (3)	C8—C9—C10—N1	−178.5 (3)
O3—S2—C16—C15	−135.2 (3)	C19—C20—C21—C22	0.8 (6)
O4—S2—C16—C15	−4.1 (3)	C23—C22—C21—C20	0.4 (6)
C19—S2—C16—C15	110.3 (3)	N4—C22—C21—C20	−179.6 (4)
O3—S2—C16—C17	38.5 (3)	C9—C10—C11—C12	−1.7 (6)
O4—S2—C16—C17	169.5 (3)	N1—C10—C11—C12	178.3 (3)
C19—S2—C16—C17	−76.1 (3)	C7—C12—C11—C10	0.4 (6)
C7—C8—C9—C10	0.0 (6)	C15—C16—C17—C18	−1.2 (6)
C11—C12—C7—C8	1.0 (6)	S2—C16—C17—C18	−174.8 (3)
C11—C12—C7—S1	−174.6 (3)	C16—C17—C18—C13	1.7 (6)
C9—C8—C7—C12	−1.2 (5)	C14—C13—C18—C17	−1.3 (6)
C9—C8—C7—S1	174.4 (3)	N3—C13—C18—C17	177.4 (4)

### Hydrogen-bond geometry (Å, º)

**Table:**

*D*—H···*A*	*D*—H	H···*A*	*D*···*A*	*D*—H···*A*
N1—H1*A*···O8^i^	0.89	2.0800	2.959 (4)	169
N1—H1*A*···O10^i^	0.89	2.4400	3.057 (4)	127
N1—H1*B*···O14^ii^	0.89	1.9300	2.796 (4)	163
N1—H1*C*···O12^ii^	0.89	2.0500	2.920 (5)	166
N2—H2*A*···O8	0.89	2.2800	3.132 (4)	160
N2—H2*A*···O9	0.89	2.3300	2.937 (5)	125
N2—H2*B*···O11^iii^	0.89	2.1300	3.015 (5)	176
N2—H2*B*···O13^iii^	0.89	2.3900	2.979 (4)	124
N2—H2*C*···O9^iii^	0.89	2.5600	3.086 (5)	119
N2—H2*C*···O10^iii^	0.89	2.0400	2.912 (5)	166
N3—H3*A*···O6^iv^	0.89	2.1000	2.957 (4)	161
N3—H3*A*···O7^iv^	0.89	2.3700	3.115 (5)	141
N3—H3*B*···O13^i^	0.89	2.0500	2.906 (4)	163
N3—H3*C*···O1*W*^i^	0.89	1.9100	2.740 (5)	154
N4—H4*A*···O5	0.89	2.1000	2.982 (4)	169
N4—H4*A*···O6	0.89	2.3900	3.071 (4)	134
N4—H4*B*···O5^v^	0.89	2.0800	2.960 (5)	171
N4—H4*B*···O7^v^	0.89	2.3800	2.988 (5)	125
N4—H4*C*···O14^iii^	0.89	1.9800	2.861 (4)	169
N4—H4*C*···O15^iii^	0.89	2.5000	3.111 (5)	126
O1*W*—H1*W*···O1^vi^	0.86 (4)	2.18 (5)	2.834 (5)	133 (5)
O1*W*—H2*W*···O12^vii^	0.85 (4)	2.19 (6)	2.940 (6)	147 (5)
C9—H9···O10^i^	0.93	2.6000	3.359 (5)	139
C9—H9···O13^i^	0.93	2.5700	3.206 (5)	126
C14—H14···O7^iv^	0.93	2.5000	3.295 (5)	143
C14—H14···O14^ii^	0.93	2.5300	3.194 (5)	128
C15—H15···O16^ii^	0.93	2.4600	3.351 (6)	160
C23—H23···O14^iii^	0.93	2.5200	3.227 (4)	133

Symmetry codes: (i) −*x*+1, *y*+1/2, −*z*+1/2; (ii) −*x*+2, *y*+1/2, −*z*+1/2; (iii) *x*−1/2, −*y*+1/2, −*z*+1; (iv) −*x*+1, *y*−1/2, −*z*+1/2; (v) *x*+1/2, −*y*+3/2, −*z*+1; (vi) *x*+1, *y*−1, *z*; (vii) −*x*+2, *y*−1/2, −*z*+1/2.
